# Single-cell study of the extracellular matrix effect on cell growth by *in situ* imaging of gene expression†
†Electronic supplementary information (ESI) available: Sequence information, *in vitro* RCA and RT-qPCR data. See DOI: 10.1039/c7sc03880a


**DOI:** 10.1039/c7sc03880a

**Published:** 2017-10-02

**Authors:** Yupeng Sun, Ruijie Deng, Kaixiang Zhang, Xiaojun Ren, Ling Zhang, Jinghong Li

**Affiliations:** a Department of Chemistry , Key Laboratory of Bioorganic Phosphorus Chemistry & Chemical Biology , Tsinghua University , Beijing 100084 , China . Email: jhli@mail.tsinghua.edu.cn

## Abstract

Cell behaviors are known to be regulated by the cellular microenvironment. Traditional cell-population based analysis methods need to separate cells from their extracellular matrix (ECM) and cannot resolve the heterogeneity of cell behaviors. Herein, an *in situ* single-cell analysis method based on rolling circle amplification was exploited to image gene expression in single cells for investigating the effect of ECM stiffness on cell growth. This method enables the simultaneous quantifying of the cell phenotype and gene expression at the single-cell level, which can help in understanding the underlying molecular mechanism of cell growth. It is found that ECM stiffness could affect cell growth *via* regulating the expression level of the cytoskeleton-assembly associated genes PFN1 and CFL1 and their co-expression pattern. Therefore, this single-cell analysis platform may facilitate us to tap into the study of “single-cell phenotypes” and elucidate the disease association of ECMs.

## Abstract 2

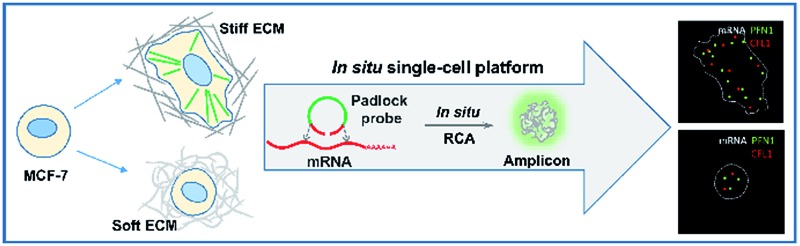
The effect of extracellular matrix stiffness on cell growth and the underlying molecular mechanism was investigated using an *in situ* single-cell imaging of gene expression method based on rolling circle amplification.

## Introduction

The extracellular matrix (ECM) not only provides a physical support for cell adhesion but also serves an important instructional role, providing biochemical and biomechanical cues.^1^–^3^ Specifically, the stiffness of the ECM plays an important role in regulating cell behaviors such as cell spreading, migration, proliferation and differentiation.^4^–^8^ Cells sense their ECM stiffness through a mechanotransduction signaling pathway which is a cellular process that translates external mechanical stimuli into intracellular biochemical signals.^3^,^9^

Cell growth and functions are regulated by gene expression programs and the disturbance of gene expression can result in many human diseases. The occurrence of cancer is not only caused by the activation of proto-oncogene and deregulation of cell-cycle control, but also abnormal defective mechanotransduction signaling may lead to tumor formation and metastatic progression.^10^ For example, a disturbance in ECM mechanics stimulates the Rho-ROCK-MLC pathway, increases cytoskeletal tension, completes a self-enforcing (positive) feedback loop and results in further increases in ECM stiffness, which can promote malignant transformation, tumorigenesis and metastasis.^11^

However, the mechanisms of mechanotransduction for cell growth on different ECMs remain incompletely understood. Recently, Mooney’s group found that the effect of substrate stress relaxation on cell spreading behavior was mediated through similar pathways as those for substrate stiffness: integrin adhesions, Rho activation, actomyosin-based contractility and nuclear translocation of YAP.^12^ However, Liu’s group investigated the gene expression of cells in response to mechanical stretching. They found that many genes related to cytoskeleton formation greatly changed after exposure to mechanical stretching (for example, PFN1 and CFL1 increased 13.0 and 1.6 folds, respectively).^13^

Recently, pillar arrays,^14^ traction force microscopy^15^ and atomic force microscopy (AFM)^16^,^17^ have been successfully applied to *in situ* determine cellular traction forces exerted by the interaction of cells and their ECMs by measuring the pillar displacement or the substrate deformations. However, detailed descriptions of the molecular mechanisms are still missed due to the lack of genetic information. Previous attempts to characterize gene or protein expression programs and investigate the molecular mechanisms were based on methods such as quantitative PCR, western blotting or RNA sequencing. Nevertheless, cells must be isolated from their cultured substrates when nucleic acids are extracted, resulting in cell–substrate interactions missed and cell status changes. Meanwhile, the overall average data lack the information of cell heterogeneity, which is widespread in biological systems, and may lead to inaccurate results.^18^–^20^

Herein, we have developed an *in situ* single-cell mRNA imaging method to investigate the effect of extracellular matrix stiffness on cell growth. In this method, the relationships of single-cell gene expressions, morphology phenotype and the effect of different ECMs were investigated by simultaneous *in situ* imaging of the cell morphology and mRNA without a complicated pretreatment process for the cells. The expression of cytoskeleton related mRNA (PFN1, CFL1 and ACTB) for cells cultured on different substrates was visualized at single-molecule levels. The multi-parameter, *in situ* single-cell study of the extracellular matrix effect on cell growth indicates the complexity and heterogeneity of cell behaviors responding to different ECMs.

## Results and discussion

### *In situ* RCA for the analysis of cytoskeleton related gene expression in single cells

**Scheme 1:**
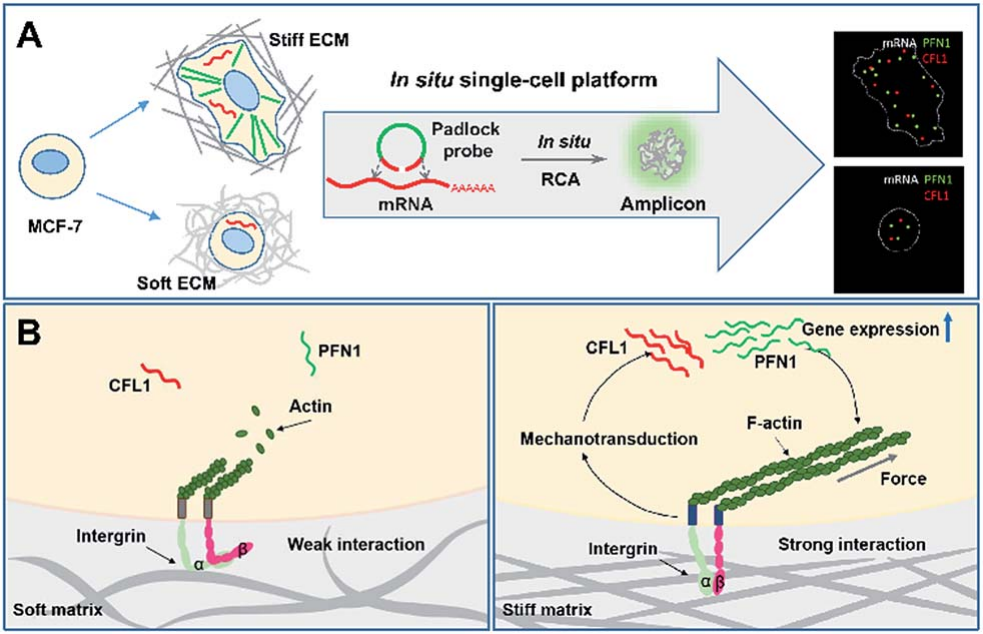
An illustration of the mechanism of cell growth on the extracellular matrix with varied stiffness *via* an *in situ* single-cell platform. (A) The effect of extracellular matrix stiffness on cell growth and the procedures of *in situ* RCA for detecting gene expression in single cells. (B) The proposed mechanisms of how the stiffness of the ECM affects the cell growth and gene expression.

Scheme 1 illustrates the effect of extracellular matrix stiffness on cell growth and the molecular mechanisms investigated *via* an *in situ* single-cell platform. As illustrated in Scheme 1A, the cells present different shapes when cultured on soft and stiff substrates, and the cells on the stiff substrate show a spindle shape and obvious stress fibers. A robust RNA imaging method based on *in situ* rolling circle amplification (RCA) has been developed to investigate the mRNA expression in single cells at the single-molecule level.^21^,^22^ Scheme 1B illustrates the proposed mechanisms of how the stiffness of the ECM affects the cell behaviors. Briefly, cells sense the ECM stiffness *via* an integrin triggered mechanotransduction pathway and the increased ECM stiffness induces specific gene expression related to cytoskeletal rearrangement (PFN1 and CFL1), forms actin filaments and promotes cell spreading and growth.^23^,^24^

### *In situ* imaging of gene expression in single cells

**Fig. 1:**
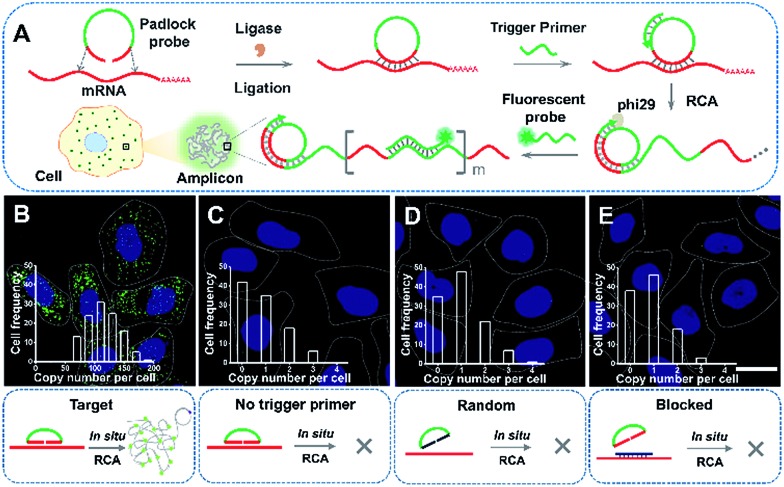
*In situ* imaging of the expression of ACTB mRNA in single cells. (A) The scheme of *in situ* RCA in single cells. Imaging of ACTB mRNA by RCA using the target padlock probe (B), without the trigger primer (C), using a random padlock probe (D), and after blocking the target site (E). The cell nuclei are stained by DAPI (blue), the RCA amplicons are hybridized with the Alexa 488-fluorescence probe (green spots), and the cell outlines are marked by a dotted line. Inset: frequency histograms of RCA amplicons per cell detected. Scale bars, 20 μm.

ACTB is a constitutive housekeeping gene which plays a critical role in F-actin formation (generating the actin monomer as the basic building unit of F-actin).^25^ A scheme of *in situ* RCA is shown in Fig. 1A. Briefly, a padlock probe was designed to recognize the target sequence of mRNA, then specifically ligate and circularize with the mRNA as the template. Next, the target mRNA could be amplified by RCA, resulting in a long DNA molecule with a large number of repeat sequences.^26^–^28^ Upon hybridization with the detection probes, the RCA amplicon would become visible as a diffraction-limited fluorescent spot.

Firstly, to demonstrate the feasibility of rolling circle amplification for RNA detection, the target sequence of ACTB mRNA was amplified *in vitro*. According to the results of the fluorescence spectra and gel electrophoresis characterization, the padlock probe can specifically recognize and effectively amplify the target sequence by RCA (Fig. S1–S3†). We further tested the performance for imaging RNAs *in situ* in single cells. As illustrated in Fig. 1B, the bright dots amplified from the target mRNA are easily distinguished from the background. A control experiment was conducted without a trigger primer which was used to initiate DNA polymerization and no obvious fluorescence signal was observed (Fig. 1C), which confirmed that the bright dots resulted from *in situ* RCA. Next, to verify the specificity of this method, a random padlock probe was used and no distinct fluorescence signal was observed (Fig. 1D). Furthermore, a blocked probe was designed to prevent the padlock probe from binding with the target sequence. Few bright dots could be detected (Fig. 1E), suggesting that the bright dot signals came from the target mRNA. The ACTB expression ranged from approximately 84 to at most 200 copy number per cell (the average was 115 per cell) and presented a normal distribution (Fig. 1B, inset; Fig. S4† shows how to quantify the copy number). The obvious variability suggested that even the same batch cells would exhibit significant cell-to-cell variation in gene expression.

### Evaluation of cell behaviors on different stiffness substrates

**Fig. 2:**
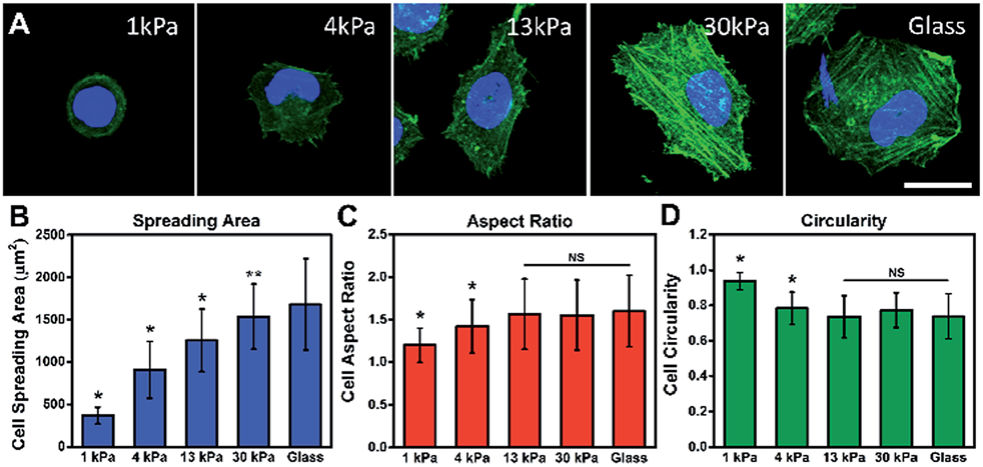
Cell morphologies on different stiffness substrates and quantitative analysis. (A) Fluorescence images of MCF-7 cells on PAAm gels with varied stiffness: 1 kPa, 4 kPa, 13 kPa, 30 kPa and glass (∼50 GPa). Cells were stained with phalloidin and DAPI to visualize the F-actins (green) and nuclei (blue) after culturing on the substrate for 12 h. Scale bar, 25 μm. Quantitative analysis of the cell morphology on the substrate: (B) cell spreading area, (C) aspect ratio and (D) circularity (*n* > 100; mean ± s.d.; **P < 0.05, *P < 0.01, NS, not significant, compare to glass).

To investigate the influence of ECM stiffness on cell morphology, MCF-7 cells were cultured on different collagen-coated polyacrylamide (PAAm) gels with different elastic moduli.^29^ The hydrogel stiffness was controlled by the ratio and the concentration of acrylamide and bis-acrylamide (details in ESI Table 1†) and the surface morphology of the hydrogel substrate is similar (Fig. S5 and S6†). The cells were cultured on gels for 12 h, then fixed and stained with Alexa 488-conjugated phalloidin and DAPI to reveal the actin filament network (green) and the nuclei (blue), respectively. The representative fluorescence images of the MCF-7 cells are shown in Fig. 2A. Obvious stress fibers (filamentous actin bundles) were seen in cells grown on a stiffer substrate (30 kPa) and a glass substrate (∼50 GPa), but not in cells grown on soft substrates (1 kPa, 4 kPa, and 13 kPa). Stress fibers, which play an important role in cellular cytoskeleton formation, can provide force for cells to sense and transmit the signal of the substrate stiffness and help cells to spread and grow.^30^

Next, to quantify the parameters related to cell morphology, the cell spreading area, aspect ratio (AR) and circularity were taken into statistical analysis. Typically, the cells on the soft substrate (1 kPa) present a round shape and smaller spreading area (400 μm^2^), while the cells cultured on the stiff substrate (30 kPa) show a spindle shape and larger spreading area (1600 μm^2^). As presented in Fig. 2B, the cell spreading area increased monotonically with substrate stiffness. Meanwhile, the aspect ratio and cell circularity (as descriptors of cell shape) were acquired from cell images using Image J. The aspect ratio of the cells was calculated as the ratio between the cell length and width.^31^ We found that the aspect ratio of the cells increased with substrate stiffness, reaching a plateau (at about 1.5) on the 13 kPa PAAm gels. Circularity, which reflects the roundness of the cells, is defined as the spreading area multiplied by 4π and divided by the square of the perimeter.^32^ Similarly, the circularity decreased with increasing substrate stiffness, reaching a plateau (at approximately 0.7) on the 13 kPa PAAm gels. The distributions of these statistical parameters are presented in Fig. S7.† Therefore, the morphological analysis suggests that ECM stiffness can affect the formation of stress fibers, cell shape and spreading.

### Timescale of gene expression during cell growth

**Fig. 3:**
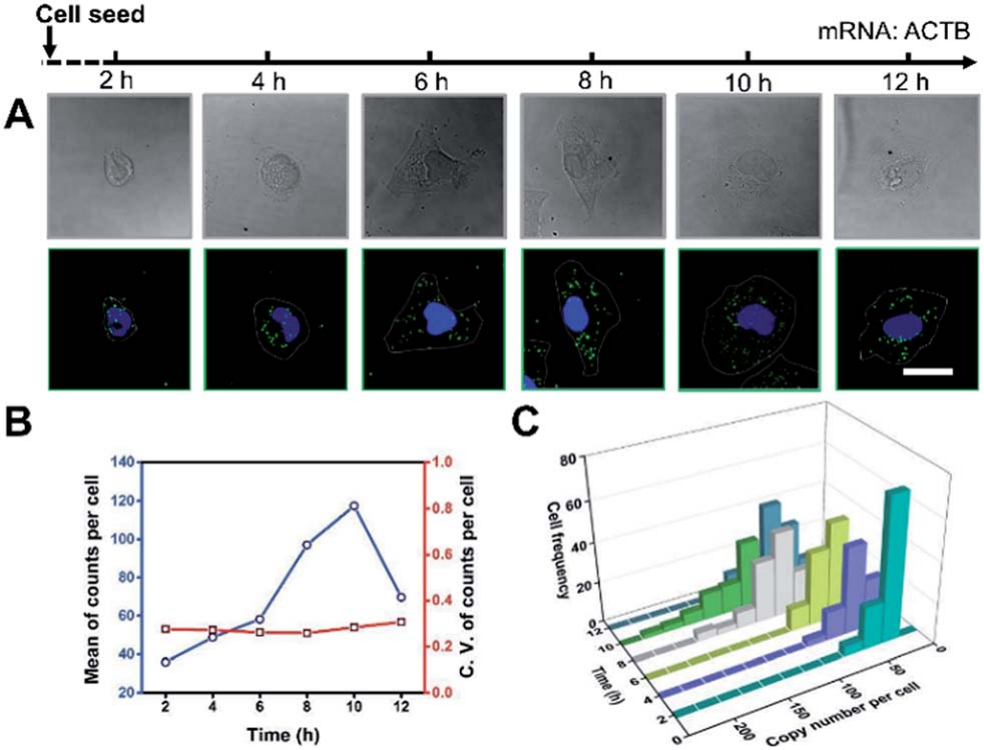
Cell morphology and ACTB mRNA expression for different culture times. (A) Imaging of cell morphology (bright field) and ACTB mRNA by *in situ* RCA (dark field). Scale bars, 25 μm. (B) The average expression level and coefficient of variation for ACTB mRNA with different incubation times. (C) Single-cell fluctuation profiles for ACTB mRNA with different incubation times (*n* > 100, C.V. = s.d./mean).

It’s a complicated process for cells to sense the ECM stiffness *via* a mechanotransduction pathway which includes sensing, signalling and gene expression to lead cell function. The timescale of these events ranges from seconds to weeks.^3^ To investigate the timescale of cell–substrate interaction, we utilized our single-cell mRNA imaging method to visualize the gene expression of cells in different culture times. As shown in Fig. 3A, the cells present varied morphologies and mRNA expression at different time points. It was found that the quantity of ACTB mRNA increased with the incubation time and reached a maximum (120 copies per cell on average) after 10 h attachment (Fig. 3B). This may be because the cell spreading process was finished and the cells tended to express less ACTB mRNA.^33^,^34^ Besides, the coefficient of variation of ACTB mRNA copy numbers per cell was more than 0.2 (Fig. 3B), indicating that the degree of dispersion for ACTB mRNA expression in different cells was high. These results emphasize the importance of single-cell mRNA detection due to cell heterogeneity. As the incubation time increased, the ACTB mRNA fluctuations evolved from a narrow and peaked histogram into a widely dispersed profile, with the average shifting to higher copy numbers (Fig. 3C). The single-cell profiling results indicate that the distribution pattern of gene expressions in single cells can be distinct at different stages and may help us to understand the procedure of cell spreading and the formation of stress fibers.

### Gene expression variation with ECM stiffness

**Fig. 4:**
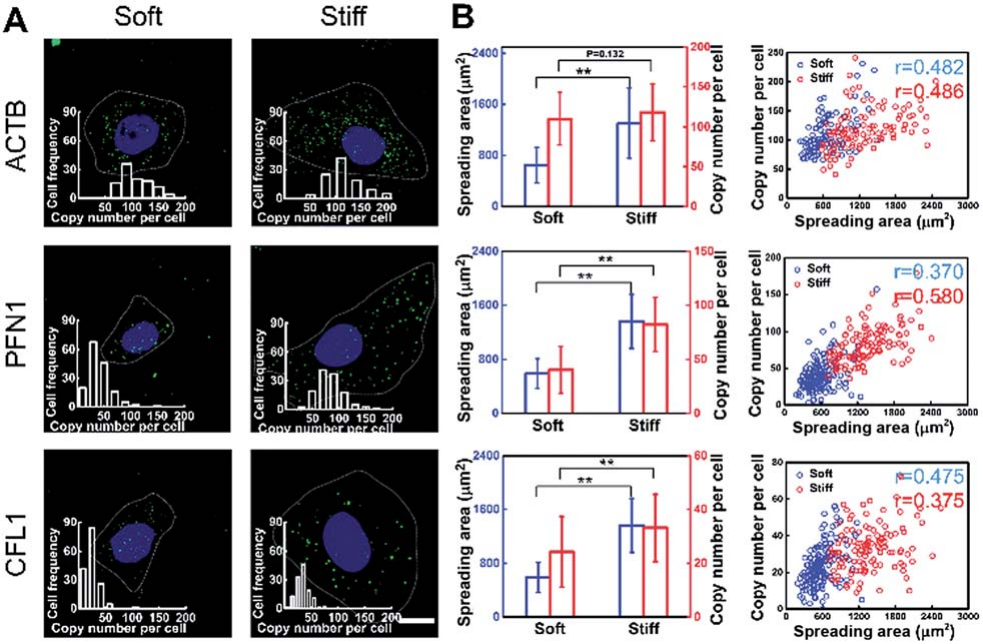
Correlation analysis of the single-cell spreading area and gene expression. (A) Images of single-cell spreading behavior and gene expression of ACTB, PFN1 and CFL1 on different stiffness substrates (4 kPa and 30 kPa). Scale bars, 25 μm. Inset: frequency histograms of RCA amplicons per cell detected. (B) The relationship of single-cell gene expression and cell spreading area for ACTB, PFN1 and CFL1, respectively (*n* > 100, mean ± s.d.; **P < 0.05, ‘*r*’ means the correlation coefficient of the single-cell spreading area and copy number per cell).

To investigate the effect of substrate stiffness on cell gene expression at the single-cell level, the expression of cytoskeleton-related genes (ACTB, PFN1 and CFL1) in cells cultured on substrates with varying stiffness was *in situ* detected. These genes play important roles in cytoskeleton remodeling which is a significant process for cell growth and metastasis.^35^ The spreading area on the soft substrate was restricted to around 600 μm^2^, while on the stiff substrate it reached around 1600 μm^2^ (Fig. 4A). Specifically, for the cells cultured on the stiff substrate, the expression of PFN1 and CFL1 was nearly two times higher than for the cells cultured on the softer substrate. However, the ACTB mRNA expression level was almost consistent (1.11 fold increase compared to the soft substrate). Generally, actins (ACTB) can be used repeatedly as the basic building units of F-actin in stress fiber forming and cytoskeleton remodeling, therefore the ACTB mRNA expression level remains basically unchanged.^36^ As a validation, we compared the mRNA expression of ACTB, PFN1 and CFL1 averaged over hundreds of cells by *in situ* RCA to those obtained from a bulk RNA quantitative measurement (RT-qPCR) performed on the same cell line. Our imaging results are in good accordance with the RT-qPCR results in general (Fig. S8†).

To infer the regulatory connections between single-cell behavior and gene expression, the single-cell gene expression and cell spreading area were *in situ* investigated simultaneously and quantitatively. A correlation coefficient is regarded as a main parameter that quantifies a type of correlation and dependence. The correlation coefficient between the cell spreading area and the copy number on the stiff substrate for genes ACTB, PFN1, and CFL1 was 0.486, 0.580 and 0.375, and 0.482, 0.370 and 0.475 on the soft substrate, respectively (Fig. 4B). Compared to the soft substrate, the correlation coefficient between the cell spreading area and gene PFN1 increased and decreased for CFL1 on the stiff substrate, while it remained constant for ACTB. The single-cell correlations of the cell spreading area and gene expression indicated that the expression variations of PFN1 and CFL1, rather than ACTB, play more important roles in the cell spreading behavior when cultured on substrates with different degrees of stiffness. As the cell spreading process involves the rapid formation of the new actin filaments and the degradation of the remaining filaments, it demands the up-regulation of the gene expression of PFN1 and CFL1. The increased expression level of cofilin (encoded by CFL1) would facilitate the depolymerization of the old actin filaments into actin monomers and profilin (encoded by PFN1) can elongate new actin filaments with the depolymerized actin monomers to facilitate the cell spreading.^37^

**Fig. 5:**
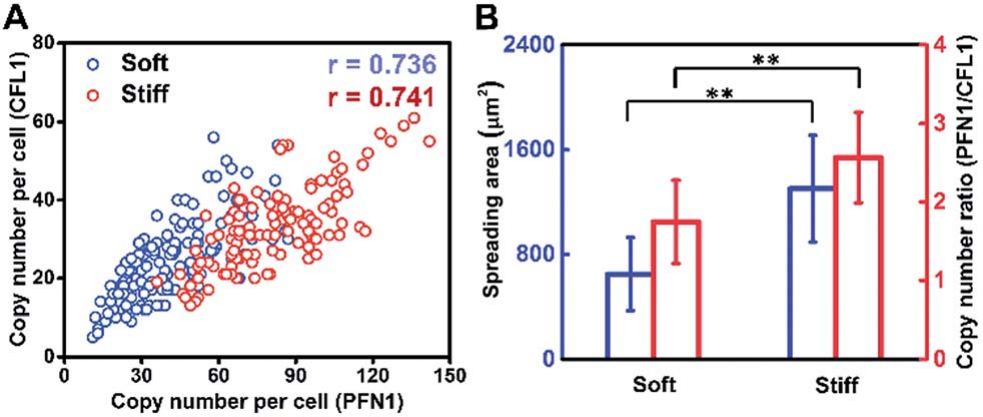
Gene co-expression patterns and correlation analysis. (A) PFN1–CFL1 expression correlation analysis in a single cell on different substrates. (B) The relationship of the single-cell spreading area and gene co-expression pattern (PFN1/CFL1) (*n* > 100, mean ± s.d.; **P < 0.05, ‘*r*’ means the correlation coefficient of the single-cell gene co-expression of PFN1 and CFL1).

Cell growth is a complicated process involving multiple genes, and we hypothesize that gene co-expression patterns have significant influences on cell behaviors on different ECMs. To test this hypothesis, we measured the single-cell co-expression patterns of key genes related to cytoskeleton rearrangement. The single-cell correlation coefficients of PFN1 and CFL1 on a stiff substrate and soft substrate were 0.741 and 0.736, respectively (Fig. 5A). The high correlation coefficients indicated that this gene pair could be regulated by a common upstream gene or directly regulate each other.^38^ Besides, it was found that the average ratios of PFN1 and CFL1 (PFN1/CFL1) in single cells were 2.570 and 1.747 for cells cultured on stiff and soft substrates, respectively (Fig. 5B). These data indicated that gene co-expression patterns could be regulated by substrate stiffness. A possible mechanism for cell growth is that ACTB is a structural gene, and PFN1 and CFL1 are regulatory genes involved in stress fiber formation, enhancing cell spreading and promoting cell growth. It is worth emphasizing that not only the expression level of PFN1 and CFL1 but also the gene co-expression patterns can regulate the assembling of the cytoskeleton in the cell spreading process.

## Conclusions

In summary, we report an *in situ* single-cell imaging method for investigating the effect of extracellular matrix stiffness on cell growth. In this method, there is no need to lyse large numbers of cells to acquire enough RNA, or to separate cells from the cultured substrate. This method can yield a simultaneous and detailed description of the single-cell gene expression profiles and morphology variance under the physical microenvironments. It is found that the increase of PFN1 and CFL1 mRNA expression levels and change in the PFN1 and CFL1 co-expression pattern, which is triggered by the stiff substrates, greatly promote the assembling of the cytoskeleton in the cell growth process. Therefore, the *in situ* mRNA imaging method can help us to understand the molecular mechanism for the influence of ECM mechanical cues on cell growth and metastasis at a deeper level. Moreover, it provides the potential to understand the mechanisms of ECM defect related diseases.

## Experimental section

### Materials and apparatus

All synthetic oligonucleotides (Table S3†) were purchased from Shanghai Sangon Biological Engineering Technology & Services Co., Ltd (Shanghai, China). The RCA detection probes were modified with Alexa488 and Cy5, and were purchased from Thermo Fisher Scientific (Waltham, USA). The salmon sperm DNA, 20 × SSC buffer (pH 7.4) and 4% paraformaldehyde in PBS buffer were purchased from Beijing Solarbio Science & Technology Co., Ltd. (Beijing, China). The deoxyribonucleotides mixture (dNTPs) was purchased from Beijing DingGuo Biotechnology Co., Ltd. (Beijing, China). Tween-20, diethy pyrocarbonate (DEPC), formamide, Triton-X100 and 3-aminopropyl triethoxysilane were purchased from Sigma-Aldrich (St. Louis, USA). TransScript one-step gDNA removal and cDNA synthesis were purchased from Transgen Biotech Co., Ltd. (Beijing, China). RiboLock RNase Inhibitor, T4 polynucleotide kinase, T4 DNA ligase, phi29 DNA polymerase, RevertAid First Strand cDNA Synthesis Kit and SYBR select master mix were purchased from Thermo Fisher Scientific (Waltham, USA). All of the solutions and deionized water used were treated with DEPC and autoclaved to be protected from RNase degradation.

### Preparation of polyacrylamide hydrogels

Briefly, PAAm gel solutions containing acrylamide monomers, cross-linker bis-acrylamide, ammonium persulphate and tetramethylethylenediamine (TEMED) were prepared. The ratio of acrylamide and bis-acrylamide and the final concentrations were varied to control the hydrogel stiffness and porosity (details in ESI Table 1†). Glass coverslips were activated by Piranha solution (H_2_SO_4_ : H_2_O_2_ = 3 : 1) and then functionalized using 3-(trimethoxysilyl)propyl methacrylate (APTES) and glutaraldehyde to facilitate the covalent attachment of the hydrogel substrates to the amino-silanated coverslips. The gel solution was sandwiched between the functionalized coverslip and a chloro-silanated glass slide to ensure easy detachment of the hydrogels.

### Preparation of PAAm gel substrates for cell seeding

To allow for cell adhesion, the surface of the hydrogel was modified with the heterobifunctional linker sulfo-SANPAH to be conjugated to a collagen I protein. The hydrogel substrate was incubated in 0.2 mg mL^–1^ sulpho-SANPAH, activated with 365 nm ultraviolet light for 10 min, washed twice with 50 mM HEPES in PBS and then incubated in 200 μg mL^–1^ of rat type I collagen in HEPES overnight at 37 °C.

### Cell culture

The MCF-7 cells were cultured in standard Dulbecco’s Modified Eagle’s medium with 10% fetal bovine serum, 1% penicillin/streptomycin, and 0.01 mg mL^–1^ human recombinant insulin. The cells were incubated at 37 °C, 5% CO_2_ and 95% air humidity. Cells were seeded on 22 × 22 mm collagen-coated glass coverslips (VWR, Radnor, USA) enclosed with PDMS as a chamber (5 mm in diameter). The varying stiffness hydrogel substrates modified with collagen were washed three times with PBS and placed in the cell culture hood for 30 min under UV light for sterilization before cell seeding. For cell seeding, the cells were plated at a proper density onto the hydrogel substrates with different degrees of stiffness, so that they had enough space to spread and didn’t contact other cells. Cells were allowed to spread for 12 h, and then were fixed and stained for analysis.

### Cell staining and image analysis

The MCF-7 cells on the substrates were fixed with 4% PFA for 10 min and permeabilized with 0.5% Triton-X100 for 5 min at room temperature, and then blocked with 1% BSA for 1 h for actin filament staining. Actin staining was performed using Alexa-Fluor 488 conjugated to phalloidin (Life Technologies, UK). After post-stain washing with PBS, the cells were mounted in 4,6-diamidino-2-5-phenylindole (DAPI, Vector Laboratories, USA) for nuclear staining. For measurements of the cell-spreading area in 2D, images of the phalloidin/DAPI-stained cells were taken using a Leica TCS SP5 inverted confocal microscope (Leica, Germany) with a 63× oil-immersion objective. Only those cells that did not exhibit any cell–cell contacts were considered in the analysis. Images of all single cells were then thresholded manually on the basis of the actin stain, and the cell spreading area was determined using Image J software.

### *In situ* visualization of mRNAs in a single-cell by RCA

As in a typical *in situ* RCA detection experiment, the hybridization of the target mRNA with the padlock probe was carried out in a volume of 20 μL solution, produced by adding 2 μL padlock probe (10 μM), 1 μL DTT (100 mM), 0.5 μL RiboLock RNase Inhibitor (40 U μL^–1^) and 4 μL yeast tRNA (10 mg mL^–1^) to 12.5 μL RNase-free water, overnight at 37 °C. Then, the sample was washed using PBS-T (DEPC-PBS with 0.05% Tween-20) three times at room temperature. The ligation process was conducted in a volume of 10 μL containing 1 μL T4 DNA ligase (5 U μL^–1^), 1 μL 10 × T4 DNA ligase reaction buffer, 0.25 μL RiboLock RNase Inhibitor (40 U μL^–1^) and RNase-free water at 37 °C for 2 h. The primer hybridization reaction was then performed with a 20 μL mixture containing 1 μL primer (5 μM), 1 μL DTT (100 mM), 2 μL 20 × SSC, 2 μL formamide, 13.5 μL RNase-free water and 0.5 μL RiboLock RNase Inhibitor (40 U μL^–1^) for 60 min at 37 °C. The RCA reaction mixture containing 1 μL 10 × phi29 DNA polymerase reaction buffer, 0.5 μL phi29 DNA polymerase, 0.25 μL RiboLock RNase Inhibitor (40 U μL^–1^), 3 μL dNTPs (10 mM) and 5.25 μL RNase-free water was then added and incubated for 120 min at 37 °C, followed with a wash in PBS-T. The hybridization of the detection probes with amplicons was conducted in a mixture of 100 nM fluorophore-labeled detection probes in 2 × SSC, 15% formamide and 10 ng μL^–1^ sonicated salmon sperm DNA at 37 °C for 30 min. The sample was washed three times using PBS-T. After being mounted with Fluoromount-G, the slides were ready for imaging.

## Conflicts of interest

There are no conflicts to declare.

## Supplementary Material

Supplementary information
