# Supplementary material for: Single-cell study of the extracellular matrix effect on cell growth by in situ imaging of gene expression
Source: Chem Sci. 2017 Oct 2;8(12):8019–24. doi: 10.1039/c7sc03880a (PMC5855132; doi:10.1039/c7sc03880a)
Supplement: Supplementary file 1 [file SC-008-C7SC03880A-s001.pdf]

Electronic Supplementary Material (ESI) for Chemical Science.
This journal is © The Royal Society of Chemistry 2017

Supporting Information

Single-cell study of extracellular matrix effect on cell growth by *in situ* imaging of gene expression

Yupeng Sun, Ruijie Deng, Kaixiang Zhang, Xiaojun Ren, Ling Zhang and Jinghong Li*

Department of Chemistry, Key Laboratory of Bioorganic Phosphorus Chemistry & Chemical Biology, Tsinghua University, Beijing 100084, China.

E-mail: jhli@mail.tsinghua.edu.cn

Table of Contents

1. Experimental Procedures S2
2. Figure. S1. Target DNA assay by RCA *in vitro*. S4
3. Figure. S2. Quantitative detection of the target sequence *in vitro*. S5
4. Figure. S3. Quantitative detection of real mRNA GAPDH by RCA *in vitro*. S5
5. Figure. S4. Identification of RCA amplicons in the fluorescence image. S6
6. Table S1. PAAm Hydrogel mechanical and swelling properties after polymerization of relative acrylamide and bis-acrylamide concentration. S7
7. Figure. S5. Fabrication of PA hydrogel substrate with varied stiffness. S7
8. Figure. S6 FT-IR spectra characterization. S7
9. Figure. S7. Distributions diagrams of cell spreading area, aspect ratio, and circularity of MCF-7 on different substrates. S8
10. Figure. S8. Expression analysis of target mRNAs in the MCF-7 cells by RT-qPCR. S9
11. Figure. S9. Quantitative analysis of mRNAs GAPDH by RT-qPCR. S9
12. Table S2. The average numbers of ACTB, PFN1 and CFL1 in the MCF-7 cells measured by *in situ* RCA and RT-qPCR. S9
13. Table S3 Oligonucleotide sequences S10
14. References S10

Experimental Procedures

Preparation of RCA amplicons *in vitro*. Firstly, the padlock probe was phosphorylated. The reaction was carried out in a volume of 20 μL containing 2 μL 10 × T4 polynucleotide kinase reaction buffer (500 mM Tris-HCl, 100 mM $MgCl_2$, 50 mM DTT, 1 mM spermidine, pH 7.6 at 25 °C), 2 μL ATP (100 μM), 13.5 μL RNase-free water, 2 μL padlock probe (100 μM) and 0.5 μL T4 polynucleotide kinase (10 U/μL) with 37 °C for 1h, followed with 65 °C for 20 min. Secondly, the ligation reaction was carried in a volume of 20 μL to acquire ring templates, which consist of 2 μL primer (10 μM, serving as a ligation template) or target DNA sequences with different concentrations, 2 μL of the phosphorylated padlock probe (10 μM), 2 μL 10 × T4 DNA ligase reaction buffer (400 mM Tris-HCl, 100 mM $MgCl_2$, 100 mM DTT, 5 mM ATP, pH 7.8 at 25 °C), 13 μL RNase-free water and 1 μL of T4 DNA ligase (5 U/μL). The reaction mixture was firstly heated at 95 °C for 5 min and cooled to room temperature for 1 h. T4 DNA ligase was then added and the ligation reaction was carried at 25 °C for 2 h. Thirdly, the ring templates were diluted with 1 × phi29 DNA polymerase reaction buffer, and added to the 20 μL RCA reaction mixture containing 2 μL the diluted ring template, 2 μL of 10 × phi29 DNA polymerase reaction buffer (330 mM Tris-acetate, 100 mM Mg-acetate, 660 mM K-acetate, 1% (v/v) Tween 20, 10 mM DTT, pH 7.9 at 37 °C), 6 μL dNTPs (10 mM for each of dATP, dGTP, dCTP and dTTP), 9.5 μL RNase-free water, 0.5 μL phi29 DNA polymerase (10 U/μL). The amplicons were generated from the RCA reaction performed at 30 °C for 1 h and terminated by incubation at 65 °C for 10 min.

Electrophoresis analysis for RCA amplicons. The RCA amplicons in 6 μl 1 × gel loading buffer were used for gel electrophoresis. Agarose was prepared with 40 mM Tris-acetate running buffer containing 40 mM Tris, 20 mM acetic acid, 2 mM EDTA (TAE, pH 8.0) to form 1% agarose-TAE sol-gel with 1 × gel red. The gel electrophoresis was performed on the prepared gel in TAE at 100 V for 30 min. After electrophoresis, the gel was visualized via ChampGel 5000 (Beijing Sage Creation Science Co., Ltd, China).

***In vitro* imaging of RCA amplicons.** To visualize the amplicons, 3 μL of the diluted RCA amplicons were deposited on aminosilane-treated slides for 30 min at 37 °C. Aminosilane-treated glass was prepared by incubating cleaned 22 × 22 mm glass coverslips (VWR, USA) for 1 min in 2% 3-aminopropyl triethoxysilane in acetone. A polydimethylsiloxane (PDMS) mask was applied to create a hybridization chamber (3 mm in diameter) on the slide. A blocking solution containing 10 ng/μL sonicated salmon sperm DNA, 2 × SSC buffer and 0.05% Tween-20 was added and the slide was incubated for 20 min at 37 °C. Washing was performed with 2 × SSC buffer once. For hybridization of the RCA amplicons, 3 μL of hybridization mixture containing 100 nM fluorophore-labeled detection probes and 2 × SSC buffer was added and incubated at 37 °C for 30 min. After hybridization, the slides were mounted with Fluoromount-G and were ready for imaging.

SEM and FTIR Spectroscopy. Hydrogel samples on coverslips were swelled in PBS overnight, then flash-frozen by plunging into liquid nitrogen. The substrates were freeze dried overnight. Then, the gels on coverslips were sputter coated with platinum. Gel surface topography was viewed in a SU-8010 scanning electron microscope (HITACHI, Japan). Attenuated total reflection Fourier Transform IR spectra (ATR-FTIR) were carried out on an UATR Two FT-IR spectrometer (Perkin Elmer, USA). Briefly, the freeze-drying hydrogel samples were put on the window and the spectra was scanned from 400 cm^{-1} to 4000 cm^{-1}.

Image acquisition and analysis. The fluorescence images of RCA amplicons were acquired using a Leica TCS SP5 inverted confocal microscope (Leica, Germany) with a 63 × oil-immersion objective. For Alexa488-labeled and Cy5-labeled probe, Ar+ (488 nm) laser and HeNe633 (633 nm) laser were used as the excitation source, and detected with a 500-535 nm bandpass filter and a 650-750 nm bandpass filter, respectively. To ensure that all RCA amplicons were imaged, images were collected as z-stacks. Stacks of images were taken with 0.15 μm between the z-slices for imaging amplicons *in vitro* and 0.2 μm between the z-slices for imaging *in situ* in cells, and combined to a single image by MIP using Image J version 1.46r software. The superbright spot supposed to be a single amplicon was distinguished from the background signal by setting the intensity threshold with Image J version 1.46r software. To determine the copy number per cell, the superbright spots were firstly marked with a pseudo color in the fluorescence images and the outline of the cell was marked out from the bright field images. The number of mRNA was determined by counting the isolated amplicon signals inside the outline of the cell.

Real-time quantitative PCR (RT-qPCR) analysis of mRNA inside cells. The cell lines MCF-7 were harvested after counting of cells and total RNA was extracted using TransZol following the manufacturer's instructions. The total RNA concentration and quality were investigated on a NanoDrop

spectrometer (ND_200, NanoDrop Technologies, USA). The cDNA samples were prepared using TransScript one-step gDNA removel and cDNA synthesis. Briefly, a total volume of 20 μL solution containing 2 μL of the total RNA (50 ng-5 μg), 1 μL anchored oligo(dT)18 primer (0.5 μg/μl), 10 μL 2 × TS reaction mix, 1 μL TransScript RT/RI enzyme mix, 1 μL gDNA remover and 5 μL RNase-free water was incubated at 42 °C for 15 min followed by heat inactivation of reverse transcriptase for 5 s at 85 °C. The cDNA samples were stored at -80 °C for future use.

qPCR analysis of mRNA was performed with SYBR select master mix according to the manufacturer's instructions on a Bio-Rad C1000TM (Bio-Rad, USA). Templates for standard curves for the different genes were created by PCR. For this PCR, the 20 μL reaction solution contained 2 μL of cDNA sample, 10 μL 2 × SYBR Select master mix, 2 μL forward primer (5 μM), 2 μL reverse primer (5 μM), 4 μL RNase-free water. The total PCR volume was 20 μl and the PCR was carried out with 2 min at 95 °C, followed by cycling 45 × (95 °C for 15 s and 60 °C for 1 min), and finished with 60 °C for 5 min. Ct values were converted into absolute GAPDH copy numbers using a standard curve from a control RNA (human GAPDH mRNA in RevertAid First Strand cDNA Synthesis Kit). A standard curve was prepared from cDNA solutions corresponding to the serially diluted solutions of human GAPDH mRNA. The volumes and components of reverse transcription and qPCR reaction mixtures were the same as those for the test samples. Obtained results are presented as the copies of mRNA per cell. The experiment was repeated three times. The copy number of target mRNAs ACTB, PFN1, and CFL1 was evaluated by referring to the expression of GAPDH mRNA using the $2^{-\Delta\Delta Ct}$ method.[1] Calculations of transcript copy numbers were based on the number of counted cells at harvest.

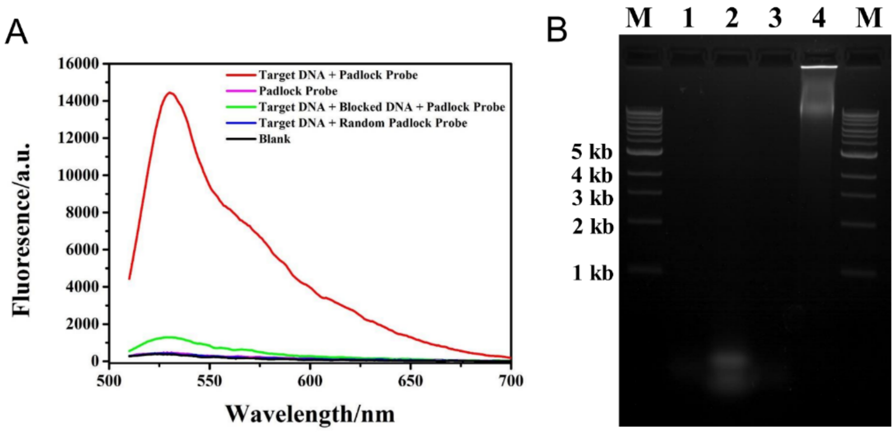

Figure S1. Target DNA assay by RCA *in vitro*. (A) Fluorescence intensity of amplification products by RCA in the absence (pink) and presence (red) of target DNA, with a random padlock probe(purple),and blocked (green). Blank means that only dye exists (black). (B) Gel electrophoresis analysis after the target DNA incubation with different probes. Lane 1: only Padlock Probe; lane 2: Target DNA + Blocked DNA + Padlock probe; lane 3: Target DNA + Random Padlock probe; lane 4: Target DNA + Padlock probe. M indicates the marker.

To demonstrate the feasibility of rolling circle amplification for RNA detection, we first did *in vitro* experiment to characterize the sensitivity and selectivity of rolling circle amplification. The amplification signal was obtained by fluorescence spectral. A series of contrast experiments was carried out, the data were shown in Figure. S1A. When the target sequence and padlock probe were presented, there generated strong fluorescence (red), while only the padlock probe involved, just faint fluorescence was produced (pink), indicating that the RCA reaction results in strong fluorescence signal amplification and low background fluorescence (shown in Figure S1B). Besides, we designed a random sequence to replace the padlock probe, added to the system contained target sequence, there generated weak fluorescence (purple), meaning that padlock probe can recognize the target sequence specifically. Further, a blocked sequence perfectly binding with the target sequence was added to the system contained target sequence and padlock probe, the fluorescence intensity showed little change (green) comparable to that of background, further comfirmed the enhanced fluorescence came from amplifying target sequence. Meanwhile, the results also were verified via agarose gel electrophoresis only padlock probe and target sequence were presented at the same time (lane 4), a bright stripe could be seen obviously near the well, in other cases, such as just padlock probe (lane 1), a random sequence (lane 3) and an additional blocked sequence (lane 2), there no distinct stripes occur, indicating that a large number of RCA products with extremely large molecular weight were produced by RCA, as shown in Figure S1C. According to the results of the fluorescence spectra and gel electrophoresis characterization, the padlock probe can specifically recognize and effectively amplify the target sequence by RCA.

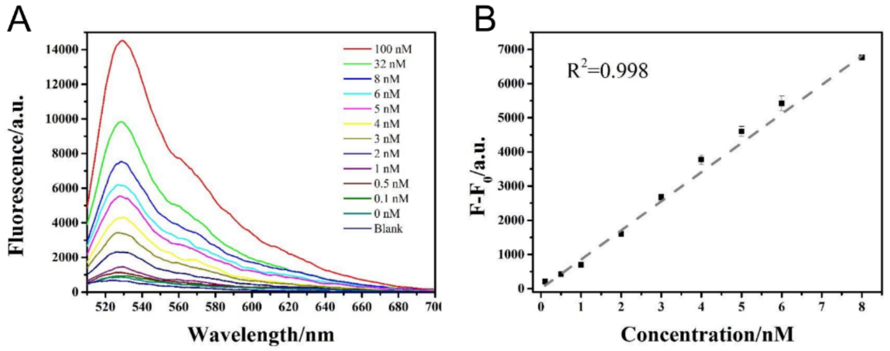

Figure S2. Quantitative detection of the target sequence *in vitro* (A) Fluorescence spectral responses to target DNA of varying concentrations ranging from 0 to 100 nM *in vitro*. (B) The corresponding calibration curve of the fluorescence intensity versus the concentration of target DNA. Error bars were estimated from three replicate measurements. The blank was deducted from each value.

The ability of this approach for quantitative detection of target DNA *in vitro* was investigated and the result is shown in Figure S2. The fluorescent intensities of RCA products with different concentration of target DNA were measured (Figure S2A), and there was a remarkable increase with the increased concentration of target DNA. A good linearity was obtained ranging from 0.1 nm to 8 nm target DNA. The correlation equation was $F - F_0 = 2.5 + 851.5\ C$ with a correlation coefficient $R^2 = 0.998$ (F and F_0 represent fluorescence intensities of amplification products by RCA in the presence and absence of target DNA; C represents the concentration of target DNA).

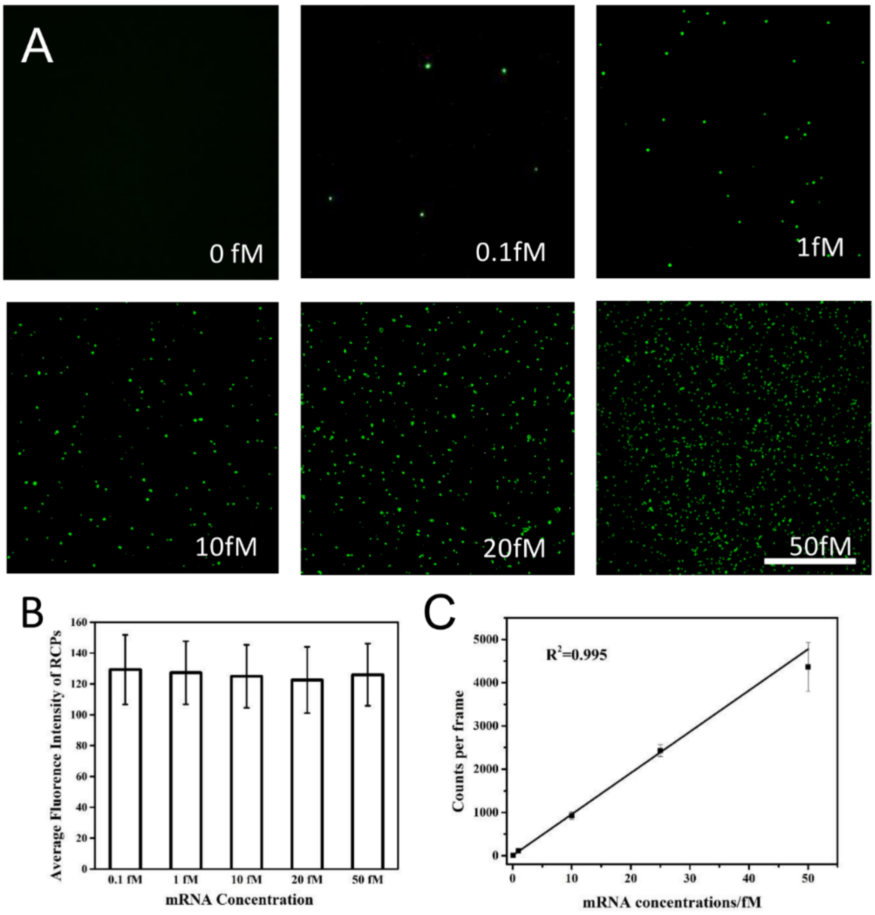

Figure S3. Quantitative detection of real mRNA GAPDH by RCA *in vitro*. (A) Fluorescence images showing labeled RCPs on the surface of coverslip with a concentration of 0 fM, 1 fM, 10 fM, 20 fM and 50 fM, respectively. (B) A diagram showing the average fluorescence intensities of RCPs dots in the images shown in (A). The error bars represent standard deviations. (C) The corresponding calibration curve of the average number of RCPs dots per image frame versus the mRNA concentration. The black line shows the linear fit to the data points. Scale bars: 25 μm.

To further confirm the ability for quantitative detection of real mRNA by RCA, GAPDH mRNA was used as target mRNA and the RCA products (RCPs) were labeled with a 22-nt fluorescent complementary oligos, resulting in bright dots when dipped into a coverslip. We acquired the RCPs by RCA using different concentrations GAPDH mRNA, respectively and then dipped into the surface of a coverslip. A series of fluorescence images of the RCPs was captured by confocal microscopy and the results were shown in Figure S3A. The RCPs conjugated with the fluorescent probe presented bright dots distributed uniformly in each image frame and these bright dots owned a similar fluorescent intensity (Figure S3B). The average diameter of the bright dots measured from the fluorescence images was approximately 1 μm. RCPs could be easily detected and identified by a conventional fluorescence microscope, making it suitable for single molecule detection. The numbers of the bright dots per image frame were counted from the images and the relationship with the GAPDH mRNA concentration was shown in Figure S3C, indicating that the numbers of the bright dots per image frame increased linearly with the mRNA concentrations. A good linearity was obtained from 0.1 fM to 50 fM of GAPDH mRNA.

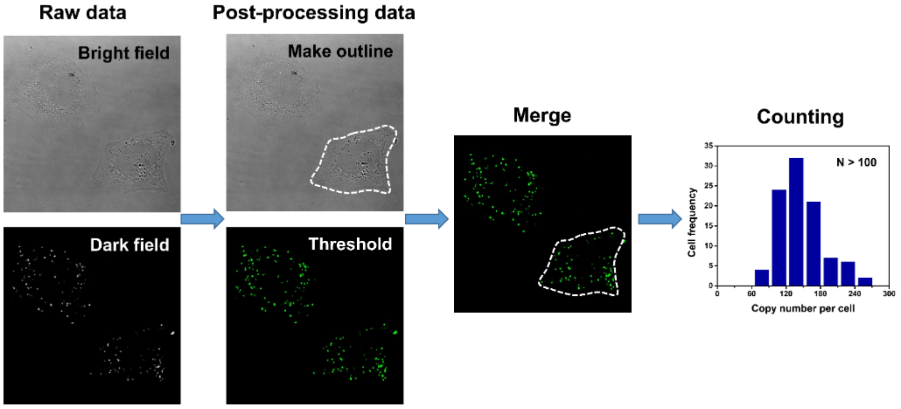

Figure. S4. Identification of RCA amplicons in the fluorescence image. The description of the procedure for counting RCA amplicons of single cells according to the previously published smFISH thresholding method.[2]

The superbright spot supposed to be a single RCA amplicon was distinguished from the background signal by thresholding the images using Image J software. The threshold value was chose based on the smFISH thresholding method, which lied in a region of plateau over which the number of spots detected is insensitive to the threshold chosen. The outline of the cell was marked out from the bright field images. The number of RCA amplicons in single cells was determined by counting the isolated fluorescence spots inside the outline of the cell using ImageJ software.

Table S1. PAAm Hydrogel mechanical and swelling properties after polymerization of relative acrylamide and bis-acrylamide concentration.[a]

Elasticity modulus (kPa)	Acrylamide (%)	Bis-acrylamide (%)	APS (%)	TEMED (%)	Q_m[b]
1	3	0.1	1	0.1	41.29 ± 3.56
4	4	0.3	1	0.1	22.31 ± 1.10
13	10	0.1	1	0.1	14.68 ± 0.71
30	10	0.3	1	0.1	11.08 ± 0.85

[a] This table presents the relative concentrations of acrylamide and bis-acrylamide and their elastic modulus and mass-swelling ratio (Q_m) after polymerization in PBS.[3] [b] The mass-swelling ratio (Q_m) is typically defined as the ratio of wet weight（M_w）to dry weight (M_d)，at least 3 times per sample were statisticsed for these measurements.

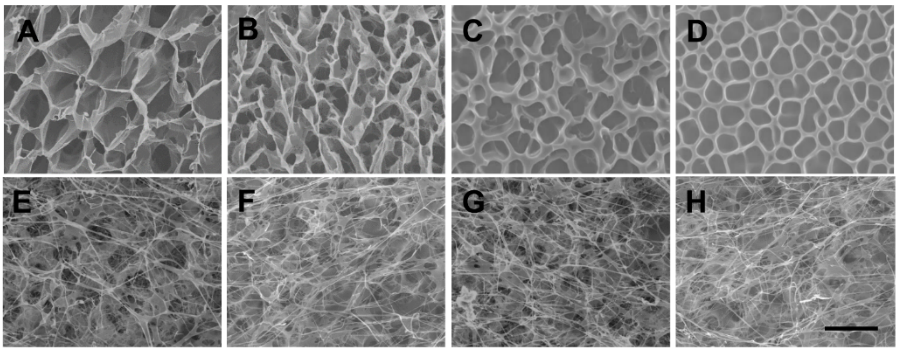

Figure S5. Fabrication of PA hydrogel substrate with varied stiffness. (A-D) SEM images of PA hydrogels of the indicated stiffness made with varying monomer : cross linker ratios. (E-H) SEM images of collagen coated PA hydrogels. (scale bars, 10 μm)

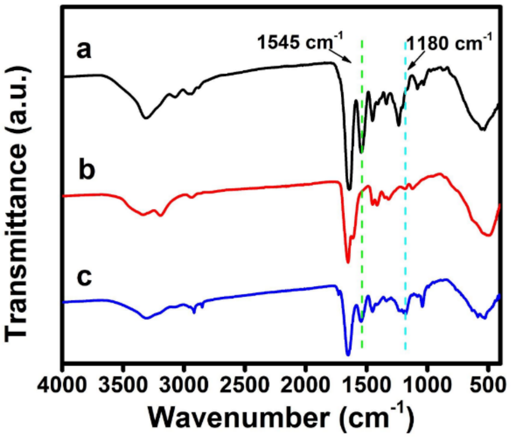

Figure S6. FT-IR spectra characterization of collagen fibers (a), PA hydrogels (b) and collagen coated PA hydrogels (c).

The characteristic bands of collagen and PAAm appeared at 1545 and 1180 cm^{-1} (curve a and b) respectively can be well observed in the corresponding spectra of collagen coated PAAm gel (curve c), indicating the successful modification of collagen onto the surface of PAAm gel.

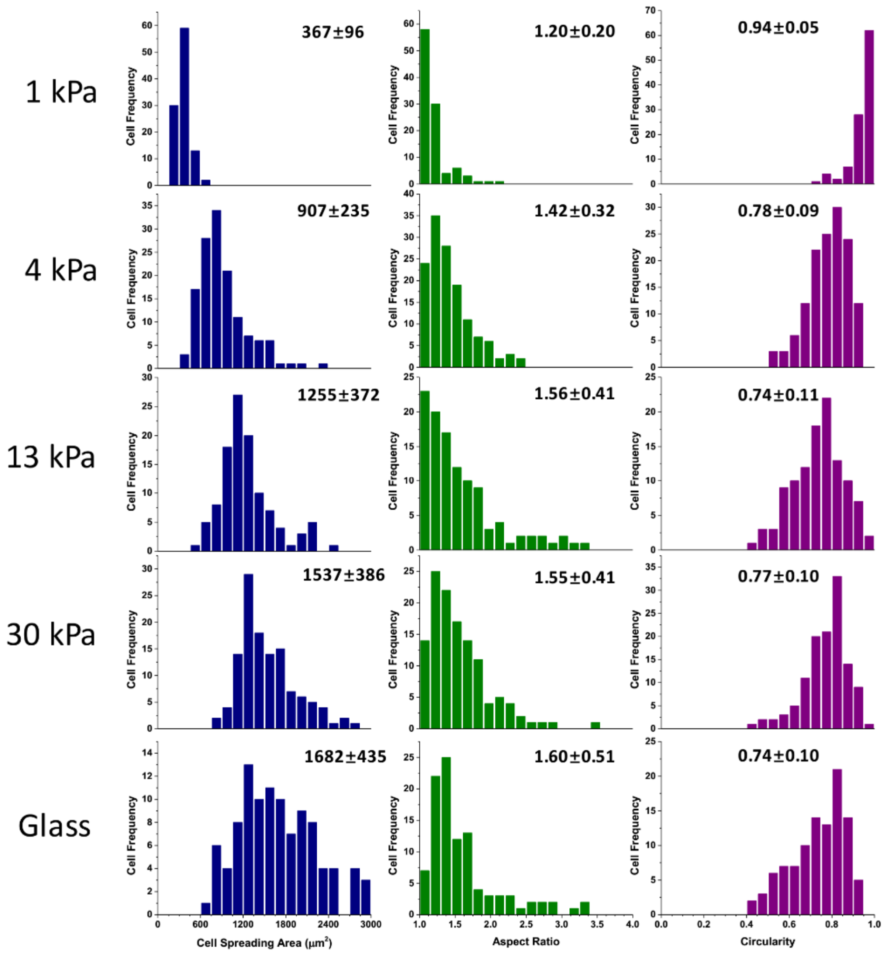

Figure S7. Distributions diagrams of cell spreading area, aspect ratio, and circularity of MCF-7 on different substrates (1 kPa, 4kPa, 13kPa, 30kPa and glass).

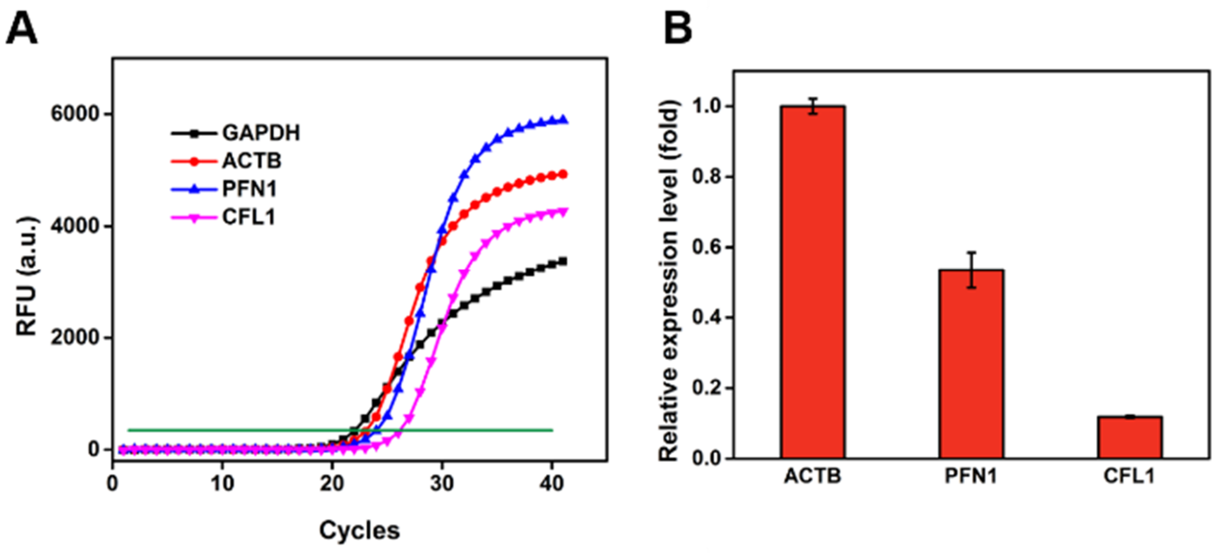

Figure. S8. Expression analysis of target mRNAs in the MCF-7 cells by RT-qPCR. (A) Real-time fluorescence curves in RT-qPCR analysis. The green horizontal line represents the threshold line. (B) The histogram of relative expression level for gene ACTB, PFN1 and CFL1. Each sample was detected in three repetitive assays.

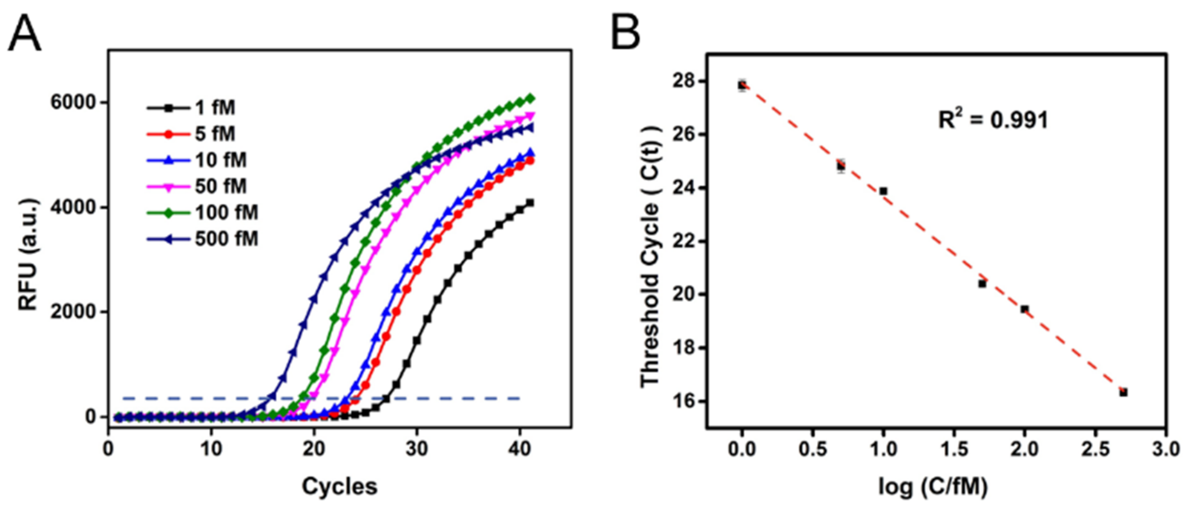

Figure. S9. Quantitative analysis of mRNAs GAPDH by RT-qPCR. (A) Real-time fluorescence curves in RT-qPCR analysis. The blue horizontal line represents the threshold line. (B) Standard curve for threshold cycle function as different concentration of mRNA GAPDH (1 fM, 5 fM, 10 fM, 50 fM,100 fM and 500 fM). Each sample was detected in three repetitive assays.

Table S2. The average numbers of ACTB, PFN1 and CFL1 in the MCF-7 cells measured by *in situ* RCA and RT-qPCR.

Target mRNA	Average number measured (copies / cell)	C_t	Average number measured by qPCR (copies / cell)	Detection efficiency (%)
ACTB	114.15 ± 30.30	22.34 ± 0.03	1128.58 ± 102.79	10.1 ± 0.29
PFN1	81.45 ± 21.55	23.25 ± 0.14	603.60 ± 50.34	13.49 ± 0.43
CFL1	33.10 ± 10.52	25.42 ± 0.04	133.76 ± 15.43	24.75 ± 0.68

Table S3. Oligonucleotide sequences

Name	Sequences (5'-3')	Description
Target sequence (GAPDH)	CCGCACCTTGTCATGTACCATCAATAAAGT (1352-1381)	Used as a model target for *in vitro* RCA
Padlock probe (GAPDH)	CATGACAAGGTGCGGTTTTTAGTGCGAACTACTACTCTCTCT TTTTAGTGCGAACTACTACTCTCTCTTTTTTACTTTATTGATG GTA	Padlock probes for *in vitro* RCA
Padlock probe (ACTB)	TGCGGTGGACGATGGAATTTTTAACTATACAACATACTACCT CAAACTATACAACATACTACCTCATTTTTTTCCGCCTAGAAGC ATT	Padlock probes for *in situ* imaging mRNA ACTB in the MCF-7 cells
Padlock probe (PFN1)	AAATGGTTTGTGTGTTTTTTCTCAATTCTGCTACTGTACTACT TTTATTGCGAACTACTACTCTCTCTATATAATGGCCCAAAAAA TA	Padlock probes for simultaneously imaging of mRNAs PFN1 and CFL1
Padlock probe (CFL1)	GTCAGCTTCTTCTTGTATTTTGCGTCTATTTTCTGGAGCCATT TTTATTGCGAACTACTACTCTCTCTTTTTTTCATGCTTGATCC CT	
Padlock probe (random)	ATCCAACCTCTTTGCTTTTTATTGCGAACTACTACTCTCTCTT TTTATTGCGAACTACTACTCTCTCTTTTTTAGTCATTTAAACTT G	Padlock probes for simultaneously imaging of mRNAs PFN1, CFL1
Triger primer (GAPDH)	AGAGAGAGTAGTAGTTCGCACT	Used as the triger primer for RCA
Triger primer (ACTB)	TGAGGTAGTATGTTGTATAGTT	
Triger primer (PFN1)	GTAGTAAAGTAGCAGAATTGAG	
Triger primer (CFL1)	ATGGCTCCAGAAAATAGACGCA	
Fluorecent probe (GAPDH)	Alexa 488-AGTGCGAACTACTACTCTCTCT	Fluorophore-labelled detection probes for visualizing RCA amplicons
Fluorecent probe (ACTB)	Alexa 488-AACTATACAACATACTACCTCA	
Fluorecent probe (PFN1)	Alexa 488-CTCAATTCTGCTACTGTACTAC	
Fluorecent probe (CFL1)	Cy5-TGCGTCTATTTTCTGGAGCCAT	
Blocking probe (GAPDH)	ACTTTATTGATGGTACATGACAAGGTGCGG	To block the binding site of mRNA for RCA detection
Blocking probe (ACTB)	CCGCCTAGAAGCATTTGCGGTGGACGATG	
GAPDH forward primer	TATGACAACAGCCTCAAGAT	Used as the primer for RT-qPCR
GAPDH reverse primer	AGTCCTTCCACGATACCA	
ACTB forward primer	CCAACCGCGAGAAGATGA	
ACTB reverse primer	CCAGAGGCGTACAGGGATAG	
PFN1 forward primer	CGAGAGCAGCCCCAGTAGCAGC	
PFN1 reverse primer	ACCAGGACACCCACCTCAGCTG	
CFL1 forward primer	CAAGGAGAGCAAGAAGGAGGAT	
CFL1 reverse primer	GTCCTTGGAGCTGGCATAAAT	

References

[1] K. J. Livak, T. D. Schmittgen, *Methods* **2001**, *25*, 402-408.
[2] S. Durdu, M. Iskar, C. Revenu, N. Schieber, A. Kunze, P. Bork, Y. Schwab, D. Gilmour, *Nature* **2014**, *515*, 120.
[3] J. R. Tse, A. J. Engler, *Current protocols in cell biology* **2010**, 10.16. 11-10.16. 16.
